# Adjustments in end-effector trajectory and underlying joint angle synergies after a target switch: Order of adjustment is flexible

**DOI:** 10.1371/journal.pone.0238561

**Published:** 2020-09-04

**Authors:** Maureen B. G. Wissing, Laura Golenia, Joanne Smith, Raoul M. Bongers

**Affiliations:** 1 Department of Human Movement Sciences, University Medical Center Groningen, University of Groningen, Groningen, The Netherlands; 2 MEDIAN Unternehmensgruppe, Medicine and Quality Management, Berlin, Germany; University of Electronic Science and Technology of China, CHINA

## Abstract

Goal-directed reaching adapts to meet changing task requirements after unexpected perturbations such as a sudden switch of target location. Literature on adaptive behavior using a target switch has primarily focused on adjustments of the end-effector trajectory, addressing proposed feedback and feedforward processes in planning adjusted actions. Starting from a dynamical systems approach to motor coordination, the current paper focusses on coordination of joint angles after a target switch, which has received little attention in the literature. We argue that joint angles are coordinated in synergies, temporary task-specific units emerging from interactions amongst task, organism, and environmental constraints. We asked whether after a target switch: i) joint angles were coordinated in synergies, ii) joint angles were coordinated in a different synergy than the synergy used when moving to the original target, and iii) synergies or end-effector trajectory was adjusted first. Participants (N = 12) performed manual reaching movements toward a target on a table (stationary target trials), where in some trials the target could unexpectedly switch to a new location (switch trials). Results showed that the end-effector curved to the switched target. Joint angles were synergistically organized as shown by the large extent of co-variation based on Uncontrolled Manifold analyses. At the end of the target switch movement, joint angle configurations differed from the joint angle configurations used to move to the original stationary target. Hence, we argue, a new synergy emerged after the target switch. The order of adjustment in the synergies and in the end-effector was flexible within participants, though most often synergies were adjusted first. These findings support the two-step framework of Kay (1988) to understand the coordination of abundant degrees of freedom and to explain adaptive actions. The flexibility in the order of adjustments of synergies suggests that the coordination of DOF emerges from self-organization.

## Introduction

A hallmark of human motor behavior is its adaptiveness, the ability to modify the action, to fit new purposes when the situation changes. Adaptiveness of actions is reflected in the adjustments to abrupt changes in the environment. Adaptiveness is observable for instance in a target switch paradigm. In this experimental paradigm a seated individual reaches with the index finger to a target (presented as a lighted target on a table or a dot on a table-mounted computer screen). Typical for this paradigm is that in some trials the target location changes unexpectedly from the original target location to a different target location on the table, at or after the moment the index finger starts to move towards the original target. In a target switch experiment, the movement path of the index finger adjusts after the target has moved to the new location and the index fingers’ path curves toward this new target location [1–6].

Goal-directed movements of the index finger require coordination of the abundant degrees of freedom (DOF) in the neuromotor system [7–9]. To illustrate this, think about the joints in the arm (i.e., DOF) that are configured in a posture that keeps the index finger (i.e., end-effector) in a certain location. There are more joint angles in the arm than minimally necessary to keep the end-effector in a certain position, implying that the joint angles can be reconfigured without moving the end-effector. Interestingly, there are also reconfigurations of joint angles that move the end-effector to a different location. Note though, because DOF are abundant they require coordination to perform goal-directed actions and effectively adjust to changes in the environment. How this coordination comes about is a key concern of studies in motor coordination.

From the dynamical systems approach to motor coordination it is proposed that the abundant DOF are coordinated in synergies (sometimes called coordinative structures [10], see Profeta and Turvey [11]). Synergies are temporary links between DOF to perform a specific task or function, emerging from interactions amongst task, organism and environmental constraints in a self-organizing manner [9, 11–15]. Switching the target to a new location changes task constraints. Therefore, this paradigm provides a means to further our understanding of the role of synergies in adaptive behavior. The current study exploits this paradigm to understand synergies through examining what happens at the DOF underlying the end-effector changes after a target switch [16, 17].

### Previous target switch studies

Previous studies examining adaptive actions following a target switch primarily focused on aspects regarding planning the end-effector movement to the switched target location. A main concern of these studies was the role of proposed feedforward and feedback mechanisms and the possible role of brain regions in those processes. In doing so, the vast majority of the studies only measured end-effector kinematics [4, 6, 17, 18]. The use of lit targets on or in a table surface, or dots on computer screens, enabled experimenters to switch target location suddenly and unexpectedly at the moment, or after, the index finger had started to move. Note that in natural situations goals or objects do not switch location instantaneously, however in current day lives people get more accustomed to such occasions, for example, when using smart phones, computer games and touch screens.

It is generally reported that in manual reaching movements, the movement trajectory of the end-effector (i.e., index finger) starts in the direction of the original target location and some time after the target has switched to the new location, curves toward the new target such that the end-effector ends up in the new target. Movement times were usually longer when the position of the target unexpectedly switched than when the target position remained stationary [2, 19–23]. The velocity profiles of the adjusted movements were characterized by double peaks and longer deceleration times [16, 20]. Earliest adjustments of the end-effector were found between 100 ms and 230 ms after the perturbation [1, 6, 17, 22, 24–26]. On the basis of a series of studies it was argued that end-effector adjustments followed from updating the initial motor program, instead of cancelling it and selecting a new motor program [1, 6, 17, 22, 24, 25, 27]. The idea proposed in those studies is that the initial motor program includes an error-corrective signal, that takes potential target position changes into account [4, 28, 29]. If the target location unexpectedly switches, the suppressed error-corrective signal is activated by efferent (feedforward) as well as afferent signals such as visual and proprioceptive feedback [5, 6, 21] to adapt the reaching movement [4, 28, 30], which results in the observed end-effector kinematics.

A few studies have addressed what happens at the level of DOF during target switch movements. Soechting & Lacquaniti [17] studied joint angle position data and muscle activation patterns of a few muscles when the target switched in a planar movement, where the arm only had two DOF, making this a non-redundant task. d’Avella et al. [16] addressed how fixed time-varying muscle waveforms (i.e., motor primitives) need to be combined to produce the muscle patterns observed after a target switch movement in which the arm was not confined to a planar movement. In this combination process the primitives could be scaled in amplitude and shifted in onset time [31]. The goal of d’Avella et al. [16] was to show how scaling parameters of amplitude and onset time of a given set of motor primitives need to vary to explain reaching movements to stationary and switched targets. Since that study asked how these scaling parameters need to be adjusted between conditions, we argue that also that paper primarily focused on the planning of the target switch movement.

### Conceptual framework

Inspired by a dynamical systems approach to motor coordination we approached the processes underlying adaptive behavior following a target switch in manual reaching movements differently than those earlier studies focusing on proposed feedback and feedforward processes or on motor primitives. To this end, next to end-effector kinematics, we measured joint angles and assessed adjustments in coordination of these DOF following a target switch. In the current paradigm the switch of a target location during the reach is interpreted as a change in task constraints. Such a change would affect the interactions among constraints and therefore the coordination of DOF in synergies that emerges on the basis of these interactions.

To understand how a change in task constraints after a target switch could affect the coordination of DOF we took the framework of Kay [32] as the starting point. In a theoretical paper Kay [32] presented an account of how degrees of freedom in the neuromotor system might be coordinated to produce goal-directed actions. The current study presents an experiment that is inspired on this account. In line with Kay [32] we assumed that coordination of DOF to perform a goal-directed action requires a two-step process. In each of these two steps constraints are active, but their function is different in each of the two steps [14, 32, 33]. In the first step of the process, constraints organize DOF in a synergy, which has the characteristics of a dynamical system that can be described with an attractor. That is, in this step DOF are assembled (i.e., linked) into a synergy, a dynamical system with lower dimensions than the number of DOF taking part in the synergy (i.e., dimensional compression). In the second step of the process, constraints affect the evolution of this dynamical system over time (i.e., affecting parameters of the attractor, determining the location of the attractor in state space or its stability), which confines the behavior of the attractor. That is, in this second step the constraints confine the synergy which results in actual behavior. In short, in our approach we assumed that constraints first act upon the DOF resulting in the emergence of a synergy, and second, constraints act to confine the synergy to produce the actual movements [32, 33]. To illustrate this, think of a reaching movement toward a stationary target in which we hypothetically propose the constraining of the DOF in two steps. In a first step, the location of the target constraints the DOF into a synergy producing a movement in a certain direction, ending the movement in a certain global location near the target. In a second step, the size of the target and its exact position further confine the synergy, resulting in the specific end-effector kinematics with which the participant’s fingertip moves to touch the target.

Exploiting the approach of Kay [32], the current paper focuses on synergies in target switch movements. Before we explain how we did this, it is relevant to briefly address our notion of synergies, because we propose to take this notion further than is usually done in a dynamic systems approach to movement coordination. Within the dynamic systems approach, also employed in the two-step framework of Kay [32], the synergy emerges based on self-organizing processes from the interactions among constraints. Based on these processes the DOF are linked in a synergy, implying that a synergy has less dimensions free to vary than the number of DOF involved [14, 32]. The synergy is understood as an emergent macroscopic order that organizes the involved DOF. Traditionally within this framework the focus has been on this macroscopic order, thereby paying less attention to the actual involved DOF and how their properties would affect the emerging order, and thus the synergy. Self-organization implies that the macroscopic order emerges from the DOF, but at the same time the macroscopic order constraints these DOF, often referred to as circular causality [34, 35]. This implies that for understanding properties of the synergy from this framework, the focus should not just be on the emergent synergy, but also on the involved DOF [36].

To do this, we take as DOF the joint angles involved in the synergy. Important, when assessing properties of synergies, we not just focus on the synergistic relations between joint angles, as is usually done, but we also examine the actual joint angles involved in the synergy. This is motivated by the notion of circular causality, which implicates that changes in relations between DOF (i.e., synergies) can not be independent of properties of the DOF (i.e., joint angles), and vice versa. Hence, although the synergy is defined as a coupling of DOF, gauging properties of synergies requires assessing synergistic organization of DOF as well as assessing how DOF are actually used. To do the latter, in the current study we analyzed clusters of joint angle configurations (see later).

A final note on how the target switch paradigm is a useful paradigm from our conceptual framework. From the proposition that synergies emerge from self-organizing processes, the stability of the synergy is an important feature. Perturbing synergies is a classic method to assess the stability of the synergy [37–40]. Since a target switch is as a perturbation, we used a target switch paradigm to assess synergies in reaching movements.

### Research questions

In the current study we applied Kay’s [32] two-step constraining process to target switch behavior. This framework assumes that DOF are always organized in synergies. Therefore, a prerequisite for applying this framework is that DOF are synergistically organized during all phases of the movement to stationary targets and to the switched targets. Therefore, the first research question asks whether DOF are synergistically organized in all conditions. Based on the wealth of studies showing that DOF are synergistically organized during a wide variety of reaching movements [41–46] we assume that our results will affirm this question.

Confirming the synergistic organization of DOF provides ground to solicit whether indeed synergies are adjusted after a target switch. Therefore, the second research question asks whether the previously reported [1, 2, 6, 16–18, 20–26] adjustment in end-effector kinematics after a target switch also involves a change in synergies. The experimental approach we took was to examine whether we found, next to the adjustments in end-effector kinematics, adjustments in coordination of joint angles after a target switch. As outlined earlier, arguably, adjustments in coordination of joint angles is indicative of changes in the synergies, based on the notion of circular causality. To assess changes in synergies we assessed the location in joint space occupied by the joint angle configurations. We determined whether the joint angle configurations used to move the end-effector to the switched target, deviated (i.e. were in a different location in joint space) from those joint angle configurations used to move the end-effector to the original, stationary target. Such an adjustment in joint angle configurations would reflect the use of a different synergy. Hence, this analysis would answer the second research question.

Assuming that the change in end-effector kinematics following a target switch is accompanied by a change in synergies, both steps (i.e., change in synergy and different confinement) in the two-step process were affected by the target switch. This provides ground to answer the third research question regarding the order in which these two processes were adjusted. Answering this research question is the main contribution of this paper. One possible scenario is that first a new synergy emerges and then this synergy is confined in a way that the end-effector moves to the switched target. In our findings this scenario would show up as that the adjustments in the joint angle configurations would take place before the adjustments at the end-effector. A second possible scenario is that first changes take place at the level of confinement of the synergy and that after these adjustments the synergy changes. In this second scenario, the analyses would show that first the end-effector movement was adjusted, after which the joint angle configurations are adjusted indicating the emergence of a different synergy.

The scenarios as outlined above assume a fixed order in which the two steps are adjusted. However, we expect the DOF to be organized in synergies that allow for variation and co-variation in organization of DOF in goal-directed actions [14, 38, 47–51]. Therefore, we should keep open the possibility that there is variation in the emergence of synergies. This leads us to a third possible scenario where the order in which synergy and end-effector kinematics are adjusted is flexible. That is, we might find that in some trials first synergies change and then this changed synergy is confined while in other trials first the original synergy to the stationary target is confined differently before a different synergy emerges. Thus, over trials we might find a flexible order of adjustments. This flexible scenario follows from the emergent and self-organizing character of the formation of synergies and the constraining processes of DOF. Because of this self-organizing character, the details of the synergy and the exact timing of when a synergy emerges cannot be predicted in detail [35, 52, 53], which would lead to a flexible order in which aspects of actions are adjusted. Hence, finding this scenario would provide a natural link between the conceptual framework of the two-step process and the experimental findings. Establishing which of these three scenarios were supported by the data, is a key contribution of the current paper. This would enable us to answer the third research question.

### Experimental approach

To answer these three research questions we employed the Uncontrolled Manifold (UCM) analysis [54–56] and variations thereof. In the UCM analysis variability of DOF over repetitions of trials is assessed with respect to the stabilization of a certain performance variable. Applied to our experiment, where we studied joint angle coordination in a reaching movement, this analysis calculates variability of joint angle configurations that does not affect the end-effector position (i.e. co-variation) and the variability in joint angles leading to deviations of the end-effector position. We argue that there is a synergistic organization of DOF if variability is structured in such a way that the variability in DOF not affecting the end-effector position (i.e. co-variation) is larger than the variability in DOF affecting the end-effector position. It has been shown consistently that in reaching movements joint angles are synergistically organized, by applying UCM analyses in different studies performed in different labs [41–46]. Applying UCM analyses would allow us to answer research question one, whether joint angles are synergistically organized in a target switch movement.

Once it has been established that DOF are organized synergistically, it needs to be determined whether a different synergy emerges after a target switch, which would answer research question two. To this end we assessed the cluster of joint angle configurations employed in a condition. This cluster has a certain size and location in joint space. In the current study we analyzed whether different clusters of joint angle configurations were used in the course of the movement when the target had switched compared to the cluster that was used when the target was stationary. This revealed whether a different synergy was used after a target switch, answering the second research question.

The final step in the analysis was to answer research question three, regarding the order of adjustments in synergies and in end-effector kinematics. Therefore, we established the moment when a different synergy emerged after a target switch. Moreover, we established the moment when the end-effector trajectory to a switched target deviated from the end-effector trajectories to a stationary target. Comparing these moments allowed us to establish according to which of the three scenarios (always synergy first, always end-effector first, or a flexible order) the DOF were coordinated in adaptive behavior following a target switch. The answer to this question enables us to relate the findings to the proposed conceptual framework.

## Materials and methods

### Participants

Twelve young adults (6 men, 6 women; mean age = 22.3 ± 1.4 years) volunteered to participate in the study. All participants were right-handed and had no motor impairments of the upper extremities. This study was conducted in accordance with the principles expressed in the Declaration of Helsinki. The protocol was approved by the ethics committee of the Center of Human Movement Sciences, University Medical Center Groningen. All participants provided written informed consent prior to the start of the experiment.

### Apparatus

Two 3020 Optotrak system sensors (Northern, Digital, Waterloo, Canada) collected the 3D position data (100Hz) of six rigid bodies, each with three LED markers, attached to the participant’s right arm and upper trunk [57]. To obtain joint angles of the index finger, one rigid body was attached to this finger in such a way that it splinted the finger to prevent motion of the inter-phalangeal joints (the finger was considered as one segment). The other five triangular shaped rigid bodies were attached to the dorsal surface of the hand, the lower arm, the upper arm, the acromion and the sternum, to obtain joint angles of the wrist, elbow, and shoulder. A standard pointer device [57] was used to digitize nineteen anatomical landmarks (tip of the 2^nd^ distal phalanx, metacarpophalangeal 2, metacarpophalangeal 3, metacarpophalangeal 5, processus styloideus os metacarpal 3, radial styloid, medial styloid, lateral epicondyle, medial epicondyle, processus spinosus 8^th^ thoracic vertebra, processus spinosus 7^th^ cervical vertebra, angulus inferior, trigonum spinae scapulae, angulus acromialis, processus caracoideus, acromioclavicular joint, sternoclavicular joint, processus xyphoideus, incisura jugularis,). These anatomical landmarks were linked to the positions of the rigid bodies and allowed the computation of the position of the fingertip and the joint angles.

**Fig 1:**
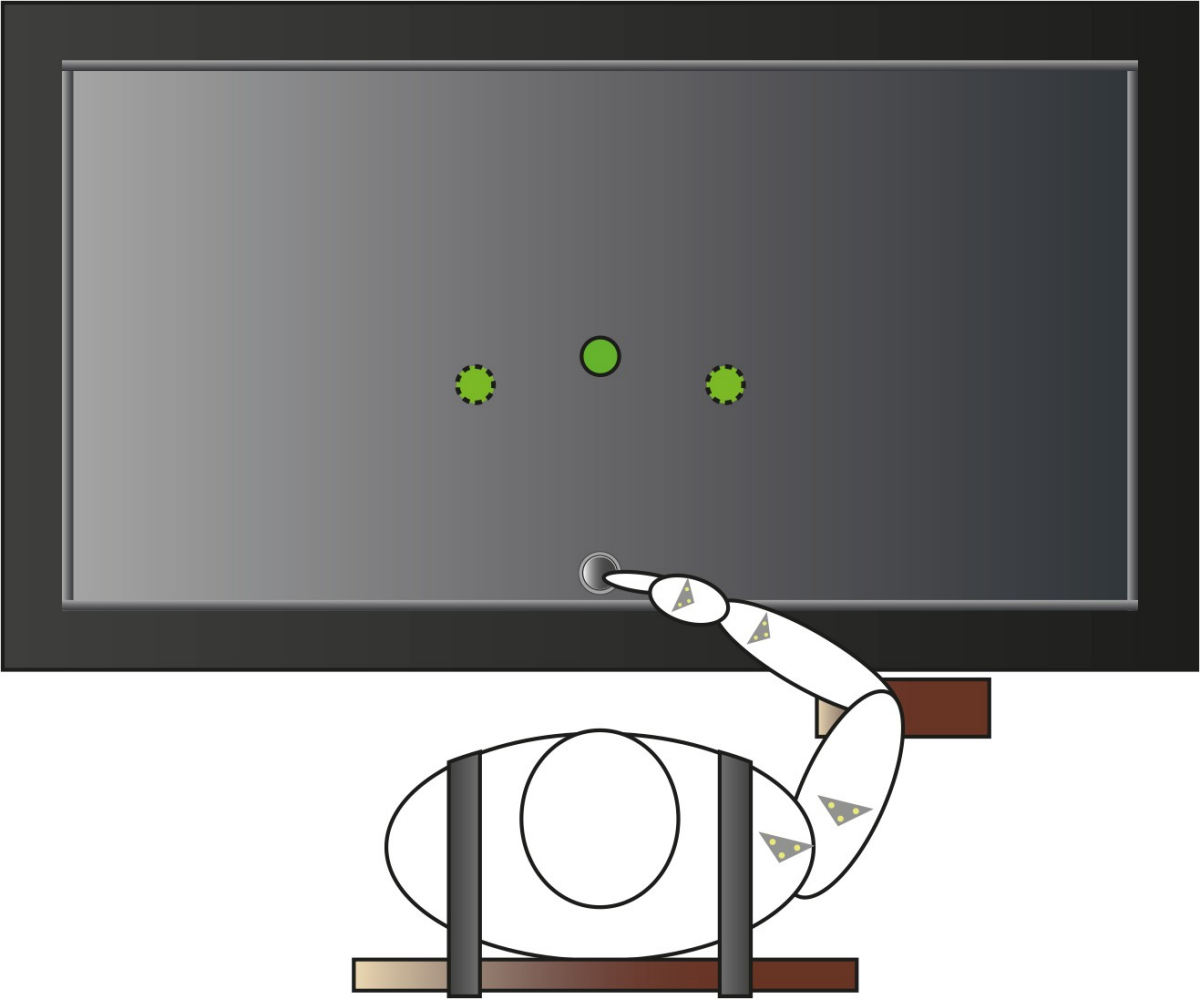
Experimental setup. A bird’s eye view of the start position of a participant, who is seated in front of a table in which the television screen is mounted. The participant is gently strapped (vertical grey bars) to the back of the chair (large horizontal brown bar) and the elbow is placed on an elbow rest (small horizontal brown bar). The index finger is located on the pressure sensor. The green circles represent the targets. The rectangle (finger) and the triangles (hand, lower arm, upper arm and shoulder) represent rigid bodies (the rigid body on the sternum cannot be seen in this schematic representation).

The task was performed at a black table (height = 72 cm) containing a large television screen (Panasonic, 62*111cm) that was horizontally mounted to present the task display. The software Presentation (Neurobehavioral systems, Berkely, CA) was used to present the task display. Participants were seated in a chair adjusted to their height facing the middle of the longer side of the screen. The back of the chair was extended with a wooden board. To prevent motion of the trunk, while allowing free movements of the shoulder and elbow, participants were gently strapped to the back of the chair. To keep the joint angle configuration at the start of the movement the same over trials, the elbow of the right arm was placed on a rest that was positioned at a comfortable height on the right side of the participant (Fig 1).

### Procedure

At the beginning of each trial, participants were instructed to place the tip of their index finger on a circular-shaped pressure sensor (2 cm diameter) while the elbow was positioned on the elbow rest. The pressure sensor was located 2 cm away from the edge of the table halfway along the long side of the screen. In all trials a green target (2 cm diameter) was presented on the screen 25 cm away from the start location at the same midline as the starting position (Fig 1, center target). Participants could initiate the reaching movement at their own convenience after hearing a beep that was emitted at a random interval of 1.0–1.5 seconds after the center target appeared on the screen. In the stationary target trials participants performed a reaching movement to the center target, which was stationary throughout the trial. Lifting the index finger from the pressure sensor at the initiation of the movement could cause the target to switch location 10 cm to the left or 10 cm to the right on a radius of 25 cm (Fig 1, dashed green circles of 2 cm diameter). The trial in which the target switched location were the switch trials. Therefore, in the switch trials participants initiated a reaching movement to the center target then, after the target switch, adapted the reaching movement to the new left or right target. Importantly, participants did not know beforehand whether a target would switch, hence the switch was unexpected and only apparent after the participants had started the movement. Participants were instructed to perform the reaching movement as fast and accurately as possible. A trial ended with holding the index finger steady on the target for a short period.

### Design

Participants started the experiment with a block of 30 stationary target trials. The results of these trials have been presented elsewhere [58] and will not be discussed in the current paper. After this block of trials, participants performed 3 blocks of 30 trials (90 trials in total) including stationary target trials and switch trials. Short breaks were allowed between blocks. In switch trials the target could either switch to the left or the right of the center target. In each block of 30 trials, each condition (stationary target, switch left, switch right) was presented in 10 trials in a pseudo-randomized order. (Note that the data for the stationary target condition in this block have also been presented in Golenia et al. [58]. Here we present new analyses of those data). The switch could occur randomly at 0 ms, 50 ms or 100 ms after lifting of the index finger from the start location, all occurring in one third of the switch trials. Although we intended to have these three switch moments, due to a programming mistake that was only discovered after the first four participants, in the trials for those four participants the target switch occurred only at 0 ms. Note that this condition was a subset of the conditions for the other participants. After checking the data we found no differences in the patterns of switches between these four and the other eight participants.

### Data analysis

For all analyses, customized data-analysis programs were developed in Matlab (MathWorks; Natick, Massachusetts). All data was filtered using a second order Butterworth filter with a cut-off frequency of 5 Hz. To determine the initiation and the termination of the reaching movement, a backward and forward search, respectively, was performed from the maximum in the tangential velocity of the tip of the index-finger. The first point detected below 5 cm/s was taken to reflect the initiation and the termination of the reaching movement, respectively. To verify these moments trials were visually checked. In addition, the shape of the velocity profiles of the switch trials were also visually checked. The velocity profile often showed two main peaks, representing the movement to the original target that was followed by a movement to the switched target. In the case that the valley in-between two peaks reached a threshold of 20% of the peak velocity, this trial was removed from the analysis because then the movement was considered to consist of two submovements. That is, the index finger almost came to a stop in these trials, resulting in a very sharp change of direction of the index finger (from a movement with a direction to that of the original target to a movement with a direction from the intermediate ‘stopping’ location to the switched target location) [59, 60]. After the data were checked, all individual trials were normalized into 100 time bins between initiation and termination of the movement.

#### Kinematic measures

To assess whether the condition (stationary target vs switch target) affected variability around the target at the end of the movement we computed the variable error around the targets for each condition separately, averaged over all participants. To establish differences in end-effector kinematics between conditions and assess whether these were in line with findings in the literature, we analyzed movement time, peak velocity and time to peak velocity. Movement time was determined as the time from movement initiation until movement termination. Peak velocity was defined as the maximum in the tangential velocity and the time to peak velocity was the time from movement initiation until the moment of maximum velocity. Additionally, for switch trials we analyzed the correction time. Correction time was defined as the time from perturbation onset until the moment of correction. To determine the moment of correction the time-normalized average lateral velocity profile of the stationary target trials and their 95% confidence interval (CI) around this average was calculated for each individual participant. In general, the lateral velocity of a switch trial started within this CI and during the trial it crossed the boundary of the CI at a side that corresponded to the location of the switched target. For every switch trial a backward search was performed to identify the last instance before the time-normalized lateral velocity crossed the boundary of the CI. This instant was taken as the moment of correction for that switch trial [1, 25]. This procedure was repeated for every switch trial to establish the correction time for each trial.

#### Uncontrolled manifold analysis

The Uncontrolled Manifold Analysis as described in previous literature [54, 55, 61] was used to determine whether DOF were synergistically organized. The analyses required the selection of elemental and performance variables. In this study the elemental variables were nine joint angles (obtained using the position data of the six rigid bodies): shoulder plane of elevation, shoulder elevation, shoulder inward-outward rotation, elbow flexion-extension, elbow pronation-supination, wrist flexion-extension, wrist abduction-adduction, index finger flexion-extension and index finger adduction-abduction. The joint angles were calculated as proposed in the International Society of Biomechanics (ISB) standardization proposal for the upper extremity [62]. The performance variable was the 3D position of the tip of the index finger. Multiple regression was used to compute the relation between changes in elemental variables and the performance variable that was used to establish the Jacobian matrix [58, 63–65]. The Jacobian matrix was used to partition the total joint angle variance into V_ucm_; variance within the null-space of the Jacobian that does not affect end-effector position, and V_ort_; variance orthogonal to the null-space that does affect end-effector position. V_ucm_ and V_ort_ were computed separately for each time bin over the trajectory, and separate for the stationary target, the switch left and switch right condition. To correct for non-normal data distribution, V_ucm_ and V_ort_ were log transformed [66]. The behavior was considered synergistic if the V_ucm_ was larger than the V_ort_ [54]. These measures were used to answer the first research question.

#### Clusters of joint angle configurations

To answer the second research question the clusters of joint angle configurations were analyzed to determine whether a different synergy emerged after a target switch. A cluster of joint angle configurations can be thought of as the region in joint space that is occupied when plotting in joint space the joint angle configurations of repetitions of trials of the same condition, at a particular instance of the time-normalized movement trajectory. We compared whether the clusters of joint angle configurations used at the beginning and end of switch trials differed from the clusters of joint angle configurations used at those moments in trials to stationary targets. To establish differences in clustering of joint angle configurations, we first computed benchmarks based on the stationary target condition. Note that the computations as explained in this paragraph were performed for each time bin (i.e., each time step in the time-normalized data series) and for each individual participant separately. The null-space and the orthogonal space of the Jacobian of the stationary target condition were calculated as described in the Uncontrolled Manifold Analysis section. Then, the joint deviation vectors for individual stationary target trials were computed as the difference between the average joint angle configuration of the stationary target condition and the joint angle configuration of an individual stationary target trial. Next, these joint deviation vectors of individual trials were projected onto the null-space and orthogonal space of the Jacobian of the stationary target condition, resulting in projection lengths within the null-space (PL_ucm_stat_, PL stands for Projection Length) and projection lengths within the orthogonal space (PL_ort_stat_). These lengths were normalized by the square root of its number of DOF [67]. Using all the trials in one condition we computed the mean projection lengths over stationary target trials and their 95% CI in the UCM (i.e., the null-space of the Jacobian) and the space orthogonal to it (CI-PL_ucm_ and CI-PL_ort_, respectively, cf. [36]). Note that the mean projection lengths were not zero because absolute values were taken when the projection lengths were computed. These CIs provided a measure for the clusters of joint angle configurations, reflecting the synergies used, in the stationary target condition.

As a next step, we projected individual switch trials onto the null-space and orthogonal space of the Jacobian of the stationary target condition, respectively, again for every time bin and participant separately. To do this, joint deviation vectors between the average joint configuration of the stationary target condition (see previous paragraph) and the joint configuration of an individual switch trial were computed. Then these joint deviation vectors were projected onto the null-space of the Jacobian of the stationary target condition and onto its orthogonal complement, resulting in the measures PL_ucm_switch_ and PL_ort_switch_ for each switch condition separately (cf. [36]). Again all projection lengths were normalized by the square root of its number of DOF. These measures provided an indication of the distance within the null-space and orthogonal space of the joint angle configuration of a switch trial compared to that of the mean of the stationary target condition. These distances can be used to assess whether different synergies are used between conditions.

To be able to check whether there were differences in synergies between the stationary target conditions and the switch conditions (i.e., whether a different synergy emerged in switch trials), the clusters of joint angle configuration between those conditions were compared. If PL_ucm_switch_ and PL_ort_switch_ fell outside the boundaries of CI-PL_ucm_ and CI-PL_ort_, respectively, we assumed that clusters of joint angle configurations between the stationary target condition and the switch conditions differed. To determine whether clusters of joint angle configurations at the beginning and end of switch trials differed from that of stationary target trials, we calculated per switch trial the percentage of instances that PL_ucm_switch_ and PL_ort_switch_ exceeded the boundaries at the first 10% and the last 10% of the movement. High percentages (between 80–100%) indicated that the clusters of joint angle configurations in the switch condition were (mostly) different than the clusters of joint angle configurations in the stationary target condition, which was interpreted as the emergence of a different synergy.

#### Timing of changes in clustering of joint angle configurations

To assess the order of changes in the synergies and the adjustments in end-effector kinematics, we first determined for each individual switch trial the moment its joint angle configurations crossed the boundary of the CI of the stationary target condition. Therefore, we established the moment at which PL_ucm_switch_ and PL_ort_switch_ exceeded the boundaries of CI-PL_ucm_ and CI-PL_ort_, respectively. To this end, we searched backward from the end of the movement for the first moment before the projection length fell inside the CI for the first time. This moment was taken as the moment the synergy had switched to a different synergy in the null-space and in the orthogonal space, respectively. The correction moment as computed for the correction time based on the lateral velocity of the end-effector (see section Kinematic measures) was taken as the moment when the end-effector trajectory to a switched target deviated from the end-effector trajectories to a stationary target. To analyze the timing between the switching at synergy level and the switching at end-effector level, we compared the moment the projection lengths fell outside the CI with the correction moment of the end-effector.

#### Statistical analyses

Separate repeated measures ANOVAs on movement time, peak velocity and time to peak velocity were performed using SPSS version 25.0 (IBM, Armonk, New York). Target condition (stationary, switch left and switch right) was the only within-subject factor in these three ANOVAs. A paired t-test was performed on correction time with switch direction (left and right) as an independent variable.

To perform the statistical analysis focusing on synergistic behavior, the V_ucm_ and V_ort_ were averaged over four equal phases (1–25, 26–50, 51–75 and 76–100% of normalized MT). A three-way repeated measures ANOVA on joint angle variance was performed, with variance (V_ucm_ and V_ort_), target condition (stationary, left and right) and phase (1–25, 26–50, 51–75 and 76–100%) as within-subject variables.

To establish whether a different synergy was used in the switch trials, we took the number of instances in which the projection lengths exceeded the CI in the first 10% and in the last 10% of the trials for each individual participant. A two-way repeated measures ANOVA on these numbers of instances with phase in the movement (begin and end) and switch side (left and right) as within-subject variables was performed.

To assess differences in timing between synergy adjustments and end-effector adjustments, we first established which of these events occurred on average earlier in time. Therefore, we conducted a repeated measures ANOVA on the first moment in normalized time at which the CI boundary was crossed. We used two within-subject variables, the level at which the boundary of the CI was crossed (joint angles and end-effector) and switch side (left and right). Second, we established for each individual trial at which of the two levels (joint angles and end-effector) the CI boundary was crossed first, which reflects the order in which CI boundary was crossed first at those levels. Therefore, we tallied the percentage of trials in which the first moment the joint angles exceeded the CI occurred earlier than the first moment the end-effector exceeded the CI, for each individual participant and switch sides separately. These percentages were compared between the switch sides with a paired samples t-test.

The significance level for all statistical tests was set at α < 0.05. With regard to the ANOVAs, if the assumption of sphericity was violated the Greenhouse-Geisser correction was applied. Generalized eta-squared, *η*^*2*^*G*, [68] was used to calculate effect sizes, and was interpreted according to Cohen’s recommendation of 0.02 for a small effect, 0.13 for a medium effect, and 0.26 for a large effect [69]. For the t-test measures of effect size (r) were used. We used Bonferroni correction when multiple post-hoc tests were performed.

## Results

Due to (partial) occlusion of the markers 6.01% of the trials were discarded. Additionally, 5.09% of the trials were discarded because in those trials movement velocity dropped below 20% of peak velocity in between peaks.

Visual inspection of the end-effector trajectories revealed that in stationary target trials the end-effector moved in an approximately straight path, whereas in the switch trials the end-effector curved to the new target location. In all conditions the variability around the target at the end of the movement was very low as evidenced by the variable errors (stationary target = 1.7 mm, switch left = 2.9 mm, and switch right = 2.5 mm).

### End-effector kinematics

**Fig 2:**
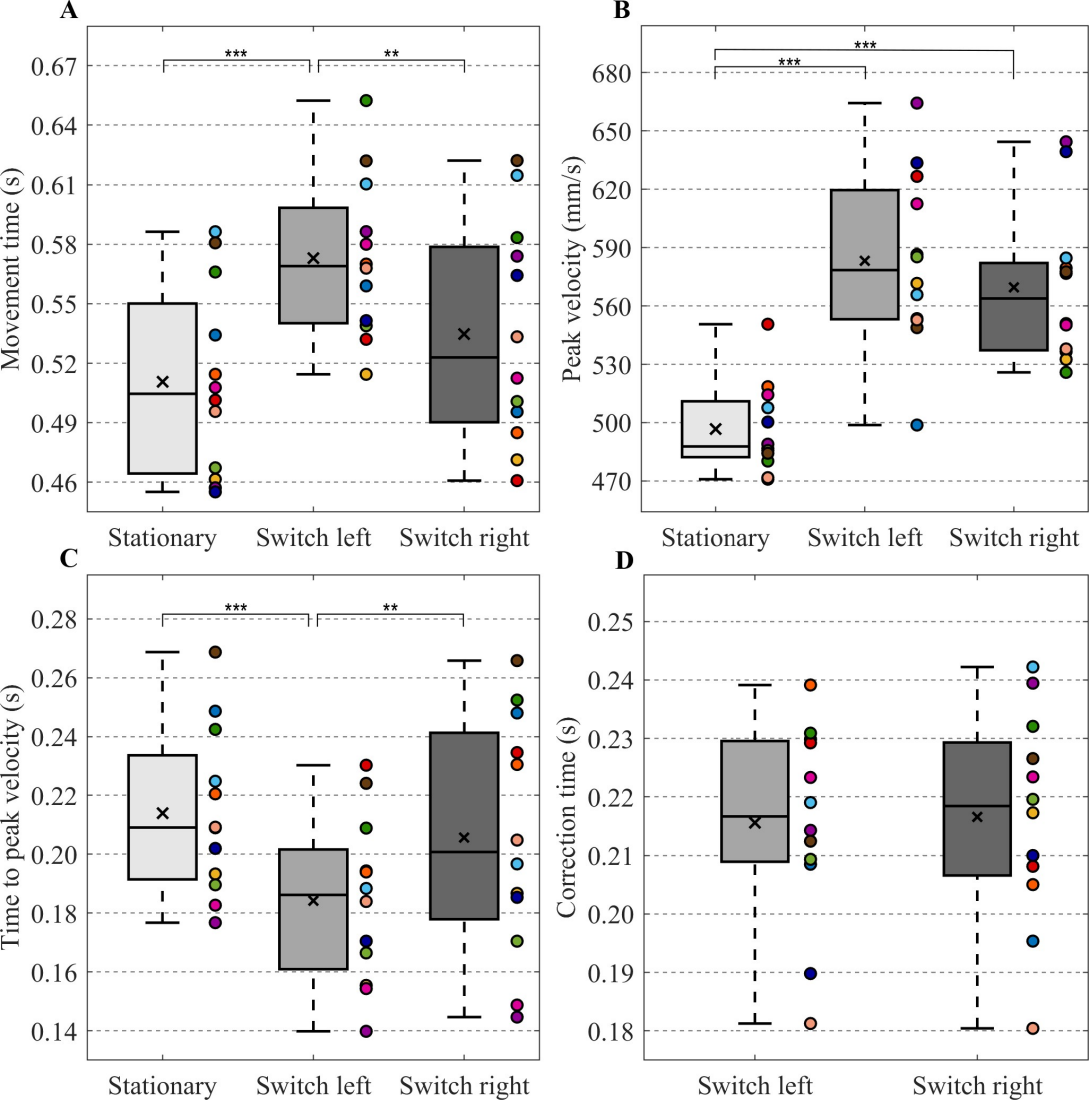
End-effector kinematics. Boxplots of the (A) Movement time (B) Peak velocity (C) Time to peak velocity and (D) Correction time. Separate boxes are plotted for the stationary target (light gray boxes), switch left (gray boxes) and switch right (dark gray boxes) conditions. A box indicates the middle 50% of the data and the lower and upper ends of the box the 25% and 75% percentile, respectively. The black x represents the mean and the black horizontal line indicates the median. Individual data is represented by colored dots positioned to the right of a box. Significant differences were indicated (** *p* < 0.01, *** *p* < 0.001).

The repeated measures ANOVA on movement time revealed a significant effect of target condition (F(2,22) = 15.06, p < 0.001, *η*^*2*^_*G*_ = 0.59) (Fig 2A). Post-hoc tests showed that movement time was longer in the switch left condition than in the stationary target condition (*p* < 0.001) and in the switch right condition (*p* < 0.01).

The repeated measures ANOVA on peak velocity demonstrated a significant effect of target condition (F(2,22) = 39.43, p < 0.001, *η*^*2*^_*G*_ = 0.78) (Fig 2B). Post-hoc tests revealed that the peak velocity in the stationary target condition was lower than the peak velocity in the switch left and switch right condition (*p’s* < 0.001).

Time to peak velocity depended on target condition (F(2,22) = 16.29, p < 0.001, *η*^*2*^_*G*_ = 0.60) (Fig 2C). Post-hoc tests demonstrated that the time to peak velocity was shorter in switch left condition compared to the stationary target condition (*p* < 0.001) and switch right condition (*p* < 0.01).

Furthermore, the paired-samples t-test on correction time did not reveal a significant difference between correction time of the switch left condition and switch right condition (Fig 2D). Note that for most participants the correction time fell in the upper range of what has been reported in the literature [1, 25].

### Synergistic organization of DOF

**Fig 3:**
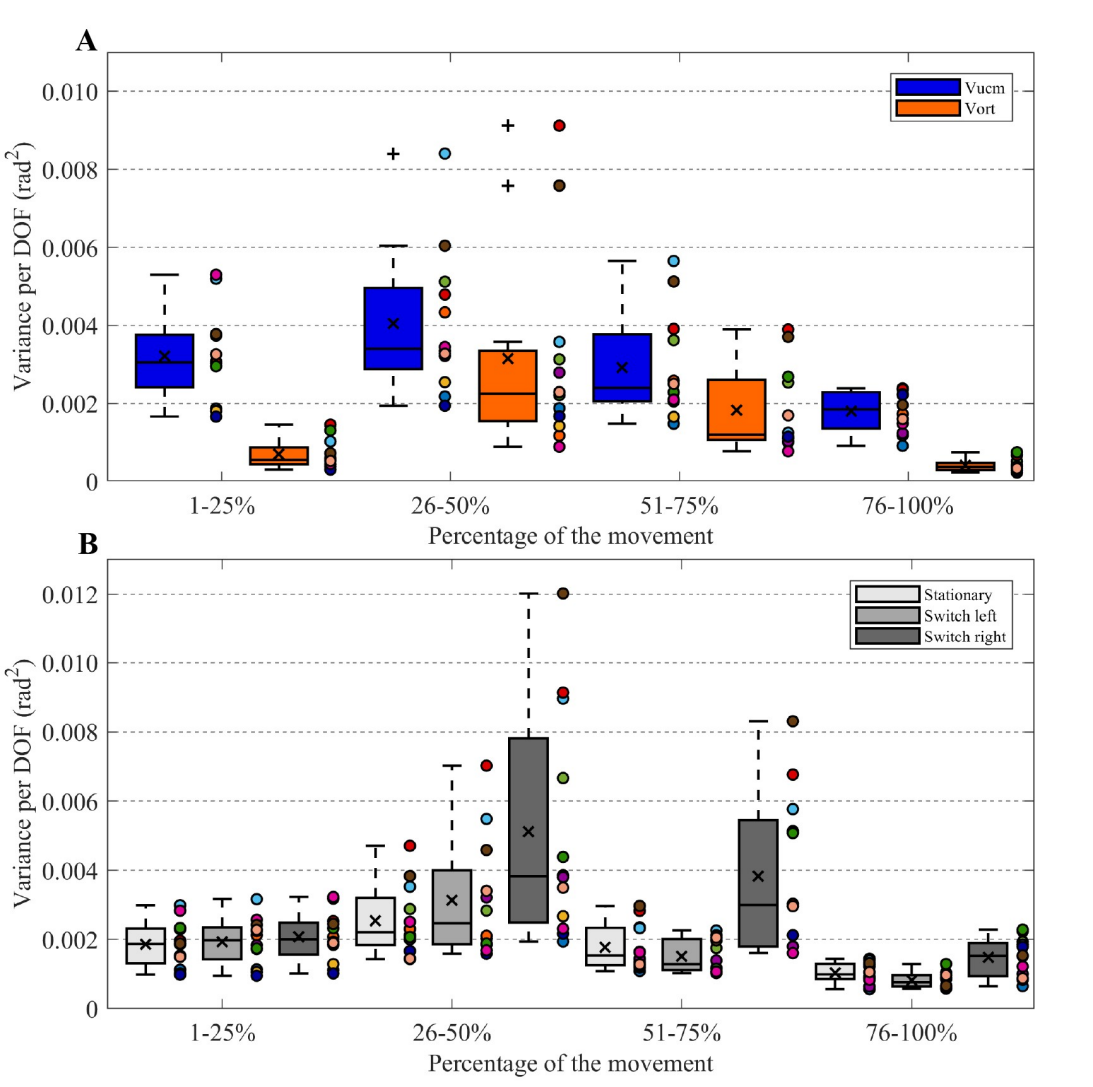
Interaction effects UCM analysis. (A) boxplots for the mean V_ucm_ (blue boxes) and V_ort_ (orange boxes) over conditions separately for four phases of the movement. (B) boxplots for the mean variance separately for the stationary target (light gray boxes), switch left (gray boxes) and switch right (dark gray boxes) conditions and the four phases of the movement. A box indicates the middle 50% of the data and the lower and upper ends of the box the 25% and 75% percentile, respectively. The black x represents the mean and the black horizontal line indicates the median. Individual data is represented by colored dots positioned to the right of a box.

The repeated measures ANOVA on the UCM variables V_ucm_ and V_ort_ showed a significant effect of variance (F(1,11) = 24.31, p < 0.001, *η*^*2*^_*G*_ = 0.24). V_ucm_ was larger than V_ort_, which indicated that DOF were synergistically organized in all conditions. Furthermore, significant main effects of phase (F(3,33) = 17.69, p < 0.001, *η*^*2*^_*G*_ = 0.32) and target condition (F(1,22) = 16.09, p < 0.001, *η*^*2*^_*G*_ = 0.18) were revealed. The interaction effect between type of variance and phase was significant (F(3,33) = 4.81, p < 0.05, *η*^*2*^_*G*_ = 0.05; Fig 3A). Post-hoc tests revealed that the difference between V_ucm_ and V_ort_ was significant in the first and last two phases (p’s < 0.01). The significant interaction (Fig 3B) between phase and target condition (F(6,66) = 9.58, p < 0.01, *η*^*2*^_*G*_ = 0.11) demonstrated that the variance in the switch right condition was higher than the variance in the stationary target condition and switch left condition for the third movement phase (p < 0.01).

### Clusters of joint angle configurations at start and end of trials

**Fig 4:**
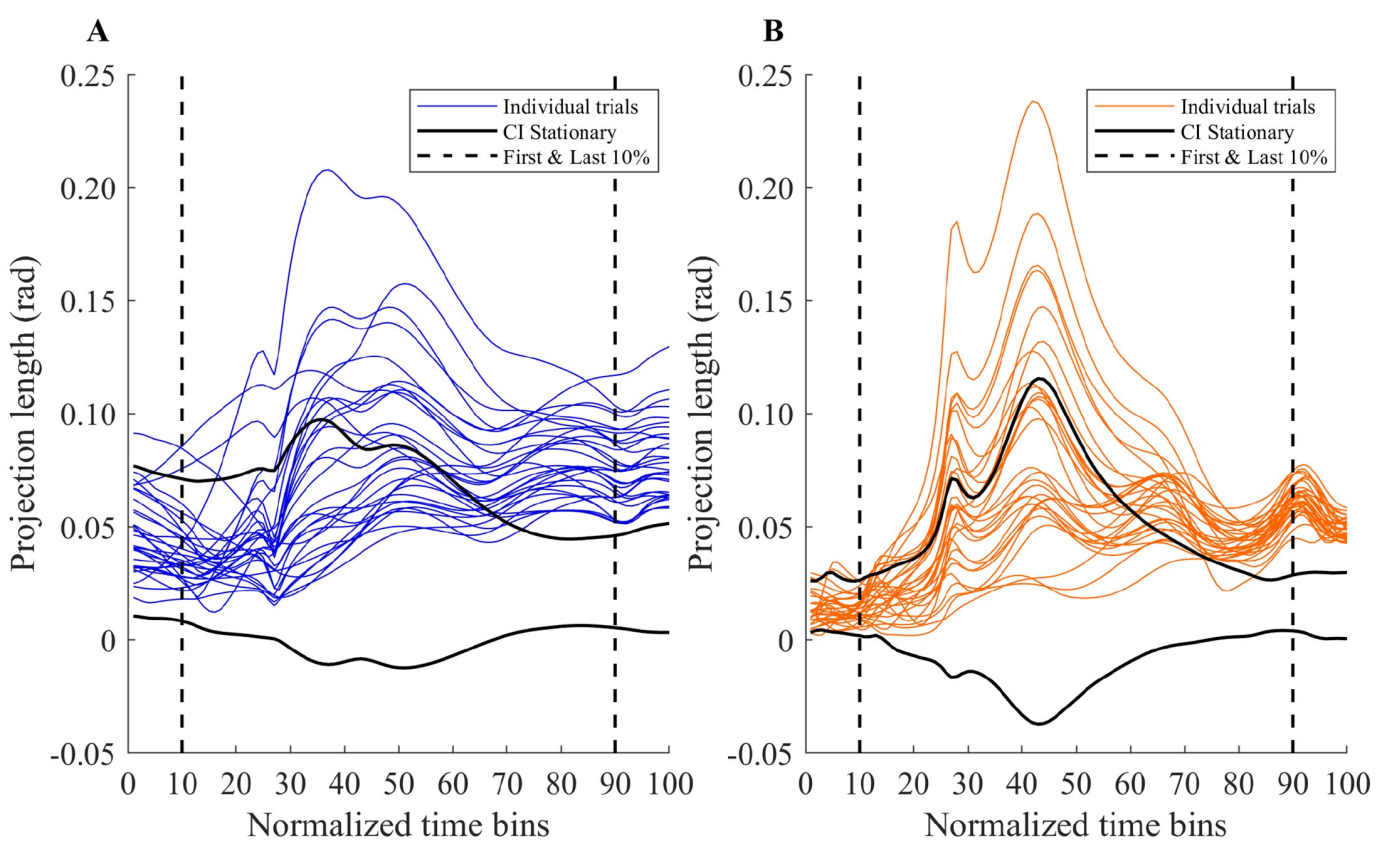
Trajectories of individual projection lengths (A) UCM and (B) ORT. An example of the projection length of individual trials in the (A) UCM (blue lines) and (B) ORT (orange lines) directions of individual switch left trials, as well as the CI’s based on the stationary target condition (thick black lines) of one participant (Participant 1 in Table 1). The dotted lines represent the first 10% and last 10% of the movement trajectory.

Now that we have established that joint angles were organized in synergies in stationary target trials and in switch trials, we can turn to the second research question asking whether synergies changed after a target switch. Therefore, we analyzed the clusters of joint angle configurations. Visual inspection of the data showed that at the beginning of the movement trajectory of switch trials the projection lengths were mostly inside the CI of the projection length of the stationary target trials. Along the trajectory of switch trials the projections lengths exceeded the CI of the stationary target trials. At the end of the movement trajectory of switch trials almost all projections lengths were outside the CIs of the stationary target conditions. This general pattern can be seen in Fig 4, where the trajectories of individual projection lengths for switch left trials (PL_ucm_switch_ and PL_ort_switch_, respectively (colored lines)) are plotted over the CIs of projections lengths for the stationary target condition (CI-PL_ucm_ (black bold lines in Fig 4A) and CI-PL_ort_ (black bold lines in Fig 4B)), for one representative participant (Participant 1 in Table 1).

**Table 1:** Percentages of switch trial projection lengths outside the CI of stationary target trials.

	Begin		End	
	% PL_ucm_switch_ or PL_ort_switch_ outside CI		% PL_ucm_switch_ or PL_ort_switch_ outside CI	
Participant	Left	Right	Left	Right
1	11	12.41	100	100
2	13.70	17.86	100	100
3	5.19	16.67	100	100
4	8.33	5.60	100	100
5	8.15	11.85	100	100
6	8.52	8.82	85.56	92.35
7	7.08	15	100	100
8	6.80	3.33	100	100
9	25.92	23.81	100	100
10	8.70	14.17	100	100
11	25	17.39	100	100
12	28.28	24.81	100	100
Mean	13.06	14.31	98.80	99.36
SEM	2.41	1.88	1.20	0.64

Per participant, over trials the mean percentage that the projection length UCM or projection length ORT of the switch trials exceeded the CI’s of the projection lengths of the stationary target trials at the first and last 10% of the movement. The mean and standard error of the mean (SEM) over participants are also reported.

Table 1 shows the overview of the percentages of instances outside CI at the first and last 10% of the movement averaged over trials for each individual participant. This table shows that in the beginning of switch trials (first 10% of movement) there are only a few instances where the projection lengths of the switch trials exceeded the boundary of the CI of the projection length of the stationary target trials. Whereas in the end of the trajectory (last 10% of movement), the projection length of the switch trials almost always exceeded the boundary of the CI of stationary target trials. The two-way repeated measures ANOVA on these instances revealed a significant main effect of phase in the movement (F(1,11) = 1802.38, p < 0.001, *η*^*2*^_*G*_ = 0.99), showing that the percentage of switch trial projection lengths outside the CI of the stationary target condition was significantly higher in the last 10% of the movement than in the first 10% of the movement. No significant differences between switch left and switch right condition were found. Additionally, Table 1 shows that participants varied in the percentage in which the projection lengths of switch trials were outside the CI of the stationary target trial in the first 10% of the movements. Note also that one participant (Participant 6 in Table 1) deviated from the general pattern at the end by showing percentages of 85% and 92% outside the confidence intervals instead of 100%.

Taken together, these results showed that in switch trials there was a transition from the use of joint angle configurations that fell within the clusters of joint angle configurations used in stationary target trials early in the reach, to the use of joint angle configurations that fell outside that cluster at the end of the movement. This implied that over the reaching movement the joint angle configurations of switch trials were different from the joint angle configurations used in stationary target trials. We interpreted this finding as that a different synergy emerged during a reach in a switch trial, which answered the second research question.

### Timing in changes of end-effector and of joint angle configurations

**Fig 5:**
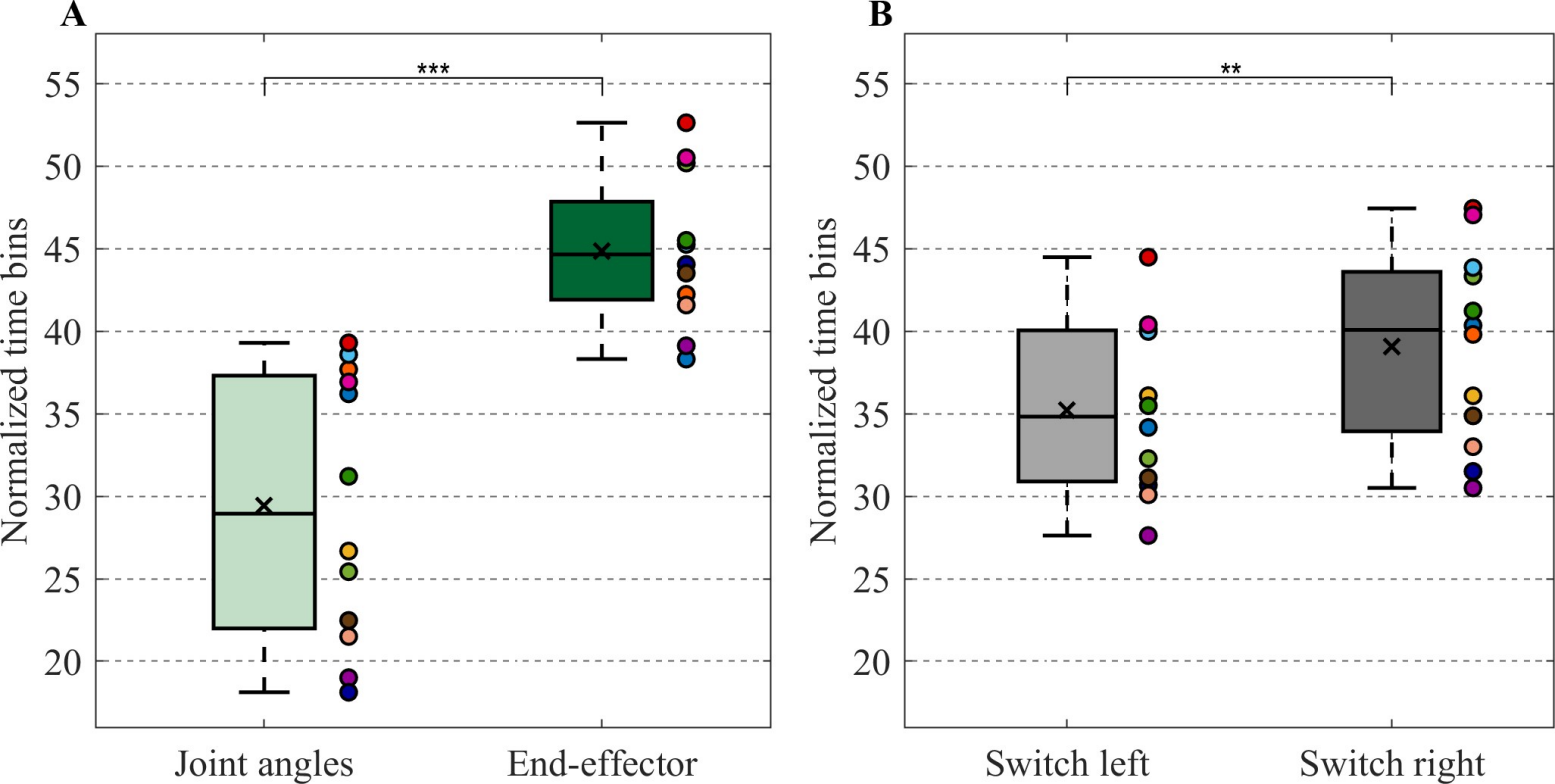
Timing of end-effector changes compared to changes in joint angles. Boxplots of (A) levels: joint angles (light green box) and end-effector (dark green box) and (B) switch side: left (gray box) and right (dark gray box). A box indicates the middle 50% of the data and the lower and upper ends of the box the 25% and 75% percentile, respectively. The black x represents the mean and the black horizontal line indicates the median. Individual data is represented by colored dots positioned to the right of a box. Significant differences were indicated (** *p* < 0.01, *** *p* < 0.001).

Since we have found that synergies were adjusted after a target switch, we now need to establish the order in which the synergy and the end-effector trajectory were adjusted, to answer the third research question. We analyzed the normalized times of when an individual switch trial crossed the boundary of the CI of the stationary target trials for the level of joint angle configurations and for the level of the end-effector trajectory. The analysis demonstrated a main effect for the level at which the boundary of the CI was crossed (F(1,11) = 47.29, p < 0.001, *η*^*2*^_*G*_ = 0.78), showing that on average the boundary of the CI was crossed earlier in the joint angles than in the end-effector, as can be seen in Fig 5A. The main effect of switch side (F(1,11) = 17.61, p < 0.001, *η*^*2*^_*G*_ = 0.18), showed that the switch occurred earlier at the left side than the right side (see Fig 5B). The interaction effect between those effects was not significant.

**Fig 6:**
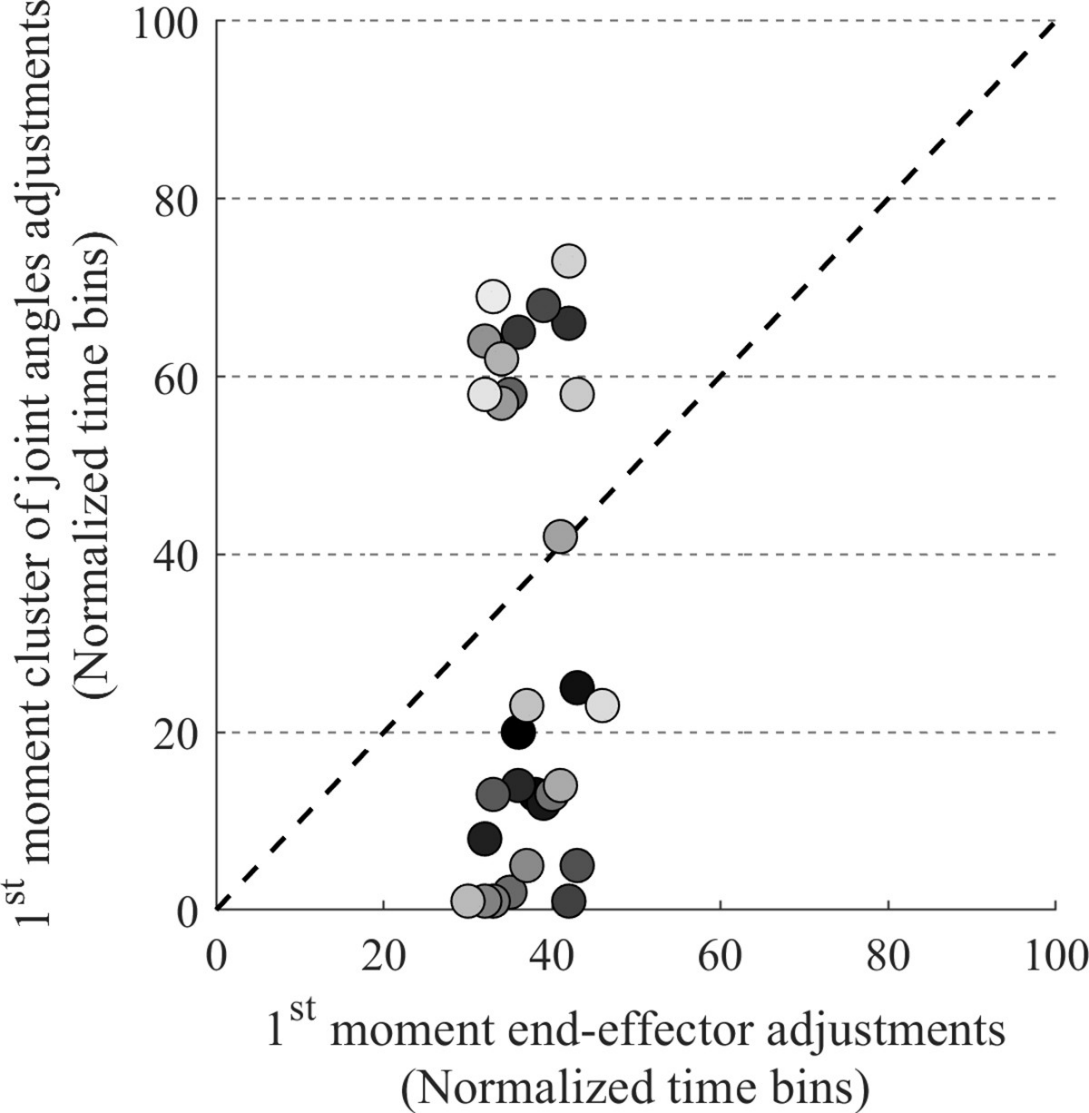
Timing of synergy and end-effector changes. The first moment of end-effector adjustments is plotted against the first moment of adjustments in cluster of joint angle configurations for switch left trials of one representative participant (Participant 1 in Tables 1 and 2). The order in which the trials were presented in the experiment is indicated by the gray shading (dark is early in the experiment and light is late in the experiment). The dashed black line represents the time where the adjustment at joint angle level is the same as that at end-effector level. Below the line are the trials in which the adjustment took place first in the cluster of joint angle configurations, whereas above the line the adjustment took place earlier in the end-effector.

The analysis just reported showed that in switch trials on average the boundary of the CI was crossed earlier at the level of the joint angles than at the level of the end-effector. Since this was an effect on the average times, it might be that the actual order in which the boundaries of the CIs were crossed could vary in individual trials. Fig 6 provides the moments an adjustment first took place (i.e., when the boundary of a CI was crossed) for the joint angle configurations and the end-effector, for switch left trials of one typical participant (Participant 1 in Tables 1 and 2). This figure shows that the order in which joint angles and end-effector were adjusted could differ between trials. That is, in some trials (the trials below the dashed black line) the initial adjustments in the joint angle configurations occurred before the initial changes in the end-effector trajectory whereas in other trials (the trials above the dashed black line) the order of the adjustments was the other way around. To assess the extent to which this occurred we examined the percentage of trials in which the cluster of joint angle configurations was adjusted before the end-effector trajectory (Table 2). All participants varied over trials in the order in which they first adjusted the joint angle configurations and they first adjusted the end-effector. None of the participants showed the adjustment always earlier in one level than in the other level. More precisely, in 8 of the 12 participants, in one or both switch sides, the percentage of trials in which the joint angle configuration was adjusted first, was 75% or smaller on average. So, although there is a tendency in the data that the switch occurred earlier in the joint angle configurations than in the end-effector, in a substantial amount of trials (i.e., around 25–30% on average) the first adjustments were made in the end-effector. Finally, the paired samples t-test revealed that the percentage of trials in which the switch occurred earlier in the joint angle configurations was higher at the left side than at the right side (t(11) = 3.27, p < 0.05, r = 0.94).

**Table 2:** Percentages of earlier switch in cluster of joint angles compared to end-effector.

	% earlier switch in cluster of joint angle configurations than the end-effector	
Participant	Left	Right
1	60	41.36
2	50	48.28
3	73.33	76.67
4	93.33	90
5	93.33	70
6	66.67	56.67
7	56.67	46.67
8	76.67	76.67
9	96.67	83.33
10	80	66.67
11	83.33	80
12	80	80
Mean	75.83	68.03
SEM	4.36	4.64

Per participant the percentage the switch in cluster of joint angles occurred earlier than the switch in end-effector of trials and the mean and SEM over participants.

Together the analyses showed that predominantly the boundary of the CI was crossed earlier in the joint angle configurations than in the end-effector. This suggests that the synergy changes earlier than the end-effector. Note, however, that in more than 1/4^th^ of the trials the boundary of the CI was crossed first in the end-effector. Together these findings demonstrated that a flexible scenario is followed in the adaptations where there is variation over trials in the order in which the synergy changes and the end-effector is adjusted to adapt behavior after a target switch.

## Discussion

The current study aimed to improve our understanding of adaptive behavior by investigating the role of synergies in coordinating DOF when a to-be-reached target switched to a new location. To do this we took the two-step process of coordinating DOF as proposed by Kay [32] as a starting point. Kay [32] argued that in a two-step process DOF are coordinated to perform goal-directed behavior. In the first step synergies emerge from the interactions of constraints and in the second step constraints confine these synergies to produce end-effector kinematics. A target switch affects constraints. To establish which of those two processes were affected by the target switch we measured joint angles and end-effector kinematics in manual reaching where the target could switch unexpectedly to a new location after the start of the reach. Note that our approach to asses joint angle configurations gauging changes in synergies was novel for the employed perspective. In the experiment the end-effector curved to the new target after a target switch, in line with what has often been reported in the literature [1–6]. Our findings showed that variability in joint angle configurations during the switching movement was primarily co-variation. This demonstrated that DOF were organized in synergies when moving to the switched target, which answered our first research question. In our second research question we asked whether a different synergy emerged after a target switch and our findings confirmed this. That is, the joint angle configurations used after a target switch differed from the cluster of joint angle configurations used to move to the stationary target, which was located at the position of the initial target in the switch trial. Although this finding in itself might not be that surprising, it opened up the possibility to ask in which order the joint angle configurations and the end-effector were adjusted after a target switch. In less than 3/4^th^ of the trials the joint angle configurations were adjusted before the end-effector, whereas in more than 1/4^th^ of the trials this order was reversed. This demonstrated that the order of changes in synergy and end-effector is flexible. We believe that finding this flexibility, in changes of synergies and end-effector kinematics, is a hallmark of coordination of DOF following from self-organizing processes. Therefore, this finding fits well in the two-step process as proposed by Kay [32]. How this framework can further our understanding of adaptive behavior will be discussed in the following. Moreover, in the remainder of the discussion we will place our findings in the context of the literature and discuss what they mean for the two-step process of Kay [32] and how they fit into a dynamic systems approach to movement coordination.

Our findings regarding adjustments in end-effector kinematics after a target switch are in agreement with the literature [2, 19–23]. Participants adjusted the trajectory of the end-effector so that it ended up in the switched target. In some conditions, movement times were longer to switched targets than to stationary targets. Note that the correction times measured at the end-effector were not particularly fast in our study, but the target switch occurred early in the movement and the distance to move was not very short. Hence, there was no pressure to adjust the movement very quickly. Moreover, we found that the movement to the left switched target lasted longer and peak velocity occurred earlier, compared to the right switched targets. This difference between left and right switch trials is probably due to the inertial anisotropy of the arm [70, 71]; the inertial anisotropy of the arm causes the acceleration to differ in different movement directions. This implied that the acceleration in the direction to the switch left target was lower than the acceleration to the switch right target. This might have resulted in the differences in the duration of the movement and the peak velocity between the switch left and the switch right target. On the basis of this idea we assumed that the underlying principles that coordinate the DOF are similar for the two switch directions, while the biomechanics produce the revealed differences in kinematics.

The current paper asked what the role of synergies was in the coordination of DOF after a target switch. Our finding that joint angles were organized in a synergy after a target switch extended earlier studies in several ways. A series of earlier studies showed that joint angles were synergistically organized during simple reaching movements to stationary targets [44–46, 72]. We showed that also during an adaptive reach after a target switch the DOF were coordinated in synergies. Our findings are in line with Mattos et al. [67] who perturbed the elbow during a goal-directed reaching movement revealing that over the trajectory of the reach joint angles were organized in synergies. Note, however, the motor equivalence analysis performed by Mattos et al. [67] assesses whether projections in null-space are larger than projections in orthogonal space whereas the current analyses examined projections in the same space but for different conditions. In addition, De Freitas et al. [41] and Golenia et al. [48, 58] examined the effect of uncertainty of the target location on the synergistic organization of joint angles and demonstrated that the amount of co-variation increased when target location could switch, compared to the situation where the target always remained stationary. However, they examined only the movements to the stationary targets and did not study the actual target switch movements as we did. In short, the finding that joint angles were coordinated in synergies during goal-directed reaches, also when a perturbation is present, is an important addition to the growing body of literature on synergies in goal-directed reaching. However, more research is necessary to establish conditions under which the change to a different synergy is permanent, as in the current study, or temporary. Therefore, future studies might explore the use of synergies when perturbing the arm, as did Mattos et al. [67], or when moving over an obstacle that perturbs the straight trajectory to the target (cf. [51]).

Interestingly, the fact that DOF are coordinated in synergies does not mean that the same synergy is used for all reaching actions. Our results showed that after a target switch a different synergy emerged that ensured that the end-effector ended in the new, switched, target location. We interpreted these results from the two-step framework of Kay [32]. We found adjustments in joint angle configurations and in the end-effector trajectory after a target switch, and, therefore, we argued that both steps of the framework of Kay [32] were involved. That is, the adjustments in joint angle configurations after a target switch reflected the emergence of the different synergy while the adjustments in end-effector trajectory reflected the changes in confinement of the synergy resulting in actual behavior. Note we took an innovative approach in this paper, assessing synergies not only at the level of the relations between joint angles, but also the actual joint angle configurations employed within a synergy. Our findings indicated that examining actual joint angle configurations might reveal how synergies adapt after perturbations, such as after a target switch.

The key question in the current paper is whether there is an order in which the two steps in the framework of Kay [32] take place in coordinating DOF. Our results support the idea that after a target switch the order in which DOF are coordinated in a synergy (i.e, step 1) and the synergy is confined to produce actual behavior (i.e., step 2), is flexible. Though, it occurred more often that first the synergy changed. This flexibility in order of adjustments of the two steps is in line with the conceptual background of the dynamical systems approach to motor coordination. According to this approach synergies emerge on the basis of self-organizing processes and for the current issue it is relevant to take into account that those processes have a stochastic component [34, 35]. Because self-organizing processes are stochastic, the exact moment a different synergy emerges is not fixed and, thus, in some trials this will be earlier, or later, than in other trials. In those cases that the different synergy emerges early in the movement trajectory, the adjustments in the end-effector will take place after the emergence of the new synergy. However, when the emergence of the different synergy happens relatively late in the movement trajectory, the change in constraints after the target switch will first affect the confinement of the original synergy. This will cause the end-effector to adjust its path before the new synergy has emerged. Since in our results we found that both situations could occur, we believe our findings support the two-step framework of Kay [32], explaining the coordination of DOF from a self-organizing perspective.

The current paper focused on synergies when addressing coordination of abundant DOF. Another notion important when aiming to understand processes underlying the coordination of abundant DOF is that of motor equivalence. Motor equivalence involves the idea that DOF reorganize when a perturbation is applied while still meeting task demands [67, 73, 74]. Motor equivalence has been studied using the analysis tools from the UCM method in experiments where the performance variable stays the same (e.g., target location of a reach or center of mass position during upright standing) while a perturbation was applied, such as a pull at the elbow during a reach [67] or a sudden displacement of the stance surface [73]. It has been shown that also when a perturbation is applied joint angles co-vary in such a way that the goal is achieved, that is, motor-equivalent joint angle configurations are used. The analyses on CIs of joint angle configurations as performed in the analyses of the current paper is inspired by motor equivalence analyses. Importantly, however, the current paper pushes the concepts underlying the UCM [56] through attempting to compare the actual synergies in terms of the clusters of joint angle configurations employed before and after a perturbation. In agreement with the studies on motor equivalence, we showed flexibility in the underlying coordinative processes to produce adaptive behavior.

We are well aware that the current study provides just a first step in understanding the processes of coordination of DOF underlying adaptive behavior from the two-step paradigm of Kay [32]. For instance, it needs to be established to what extent the current conclusions are influenced by the actual size of the target switch. That is, it might be that a small shift in target location does not require the emergence of a different synergy, whereas a larger target shift does. Moreover, in the situation that the switched target location is further away from the original target the perturbation of the interaction among constraints might be stronger, which might result in the immediate emergence of a new synergy instead of sometimes first adjustments in the confinement of the original synergy. In addition, in the current study we have not specifically made a distinction between changes in the null-space and in the orthogonal space. However, it might be that particular changes in task constraints evoke particular adjustments in either space. Finally, we choose to limit our analyses to assess coordination in DOF with UCM related measures. Alternative analyses techniques like PCA [75, 76] might reveal different aspects of coordination of DOF during adaptive behavior, hence, this deserves further attention. The current paper attempted to shift focus from studies on end-effector level to underlying coordination processes of DOF. Moreover, we aimed to extend the notion of synergy from a dynamical systems perspective to motor coordination by including the actual joint angle configurations and not just sticking to the level of relations between joint angles. Given that we identified changes in coordination of joint angles as DOF after a target switch, this level of study deserves further attention in order to advance our understanding of adaptive behavior. In the future, such an approach might also be helpful in understanding why in some trials participants used a strategy with two submovements (i.e., trials that we have not analyzed). The methodology currently employed does not allow us to analyze these trials in the same framework.

In conclusion, the current study focused on the role of synergies in producing adaptive behavior when a to-be-reached target unexpectedly switched. Our main findings were that joint angles were organized in synergies during the switching movement. Not only end-effector patterns changed after a target switch but also underlying synergies coordinating DOF. Interestingly, we found flexibility in the order in which the first adjustment could be observed in the synergy or in the end-effector. Although this order could vary within participants, in most cases the synergy changed first and then the end-effector. These findings support the two-step framework of Kay [32] to understand the coordination of abundant DOF to produce goal-directed adaptive actions. In line with this framework, the results suggest that DOF are coordinated in synergies based on self-organizing processes and that the characteristics of adaptive behavior follow from these processes.
